# Research on the dynamic evolution mechanism of disruptive technology based on the BERTopic model and Hidden Markov Model: A case study of industrial Internet technology

**DOI:** 10.1371/journal.pone.0319924

**Published:** 2025-04-17

**Authors:** Heng Yang, Sheng Chen, Xin Yang

**Affiliations:** 1 School of Public and Administration, Chongqing University, Chongqing, China; 2 School of Computer Science and Engineering, University of Electronic Science and Technology of China, Sichuan, China; Ataturk University, TÜRKIYE

## Abstract

**Objective:**

The development of key technologies for the Industrial Internet is a major concern for countries worldwide. This paper aims to comprehensively understand the technology of the Industrial Internet by analyzing its current application status and trends. It will dynamically examine the key technologies and development trends of the Industrial Internet, providing a valuable reference for technological advancements in this field.

**Methods:**

This paper analyzed global patent data in the field of the Industrial Internet from 1965 to 2023. The paper applied the BERTopic model and the all-MiniLM-L6-v2 model to extract and vectorize topics related to industrial internet technology from patent texts. Based on the theory of Internet governance, the paper categorizes the topics into four categories. The paper then established the Hidden Markov Model (HMM) to investigate the evolutionary mechanism of technological topics. The paper utilized the newly divided topics as hidden states and the number of patent applications as observed states in the Hidden Markov Model (HMM).

**Results:**

Industrial internet technology encompasses five research directions. The physical layer focuses on interconnection platforms for equipment, as well as devices for the storage and monitoring of liquids and gases. The logical layer involves remote control systems for industrial equipment, while the data layer focuses on data processing and information services. The interaction layer included modular image processing and control methods. Among these types of technologies, the data layer technologies were the most developed and also contributed to the advancement of interaction layer technologies. The physical layer technologies were relatively more developed, while the logical and interaction layer technologies were relatively less developed.

## 1. Introduction

The industrial Internet, as a key driving force for the digitalization and intelligence of the manufacturing industry, has been highly valued by countries around the world. Countries have formulated relevant strategies and policies to promote the development and application of the Industrial Internet. Examples include the United States’ “Advanced Manufacturing Partnership Program,” China’s “Made in China 2025,” Germany’s “Industrial 4.0 strategic plan,” the United Kingdom’s “British Industry 2050 strategy,” France’s “New Industrial France Plan,” Japan’s “Super Intelligent Society 5.0 strategy,” South Korea’s “Manufacturing Innovation 3.0 plan,” and many others. Globally, competition in the field of industrial Internet is also intensifying. Manufacturing powerhouses, such as the United States and Germany, continue to increase policy support and financial investment to maintain their technological advantages and market share [1]. In 2020, the projected value-added scale of the Industrial Internet in the United States is $885.84 billion, making it the global leader in this sector. China followed with $566.456 billion in value-added, which is greater than the combined value added of Japan and Germany [2]. It can be predicted that the Industrial Internet will become an important field for countries to compete in the future.

To gain a comprehensive understanding of the current development status and future trends of industrial Internet technology, it is necessary to analyze its patents. A patent is a direct reflection of technological innovation. It can reveal the core content, development direction, and competitiveness of the technology. Some studies have reviewed patent texts on smart manufacturing technology [3]), edge computing for Industry 4.0 [4], and IoT technology [5,6]. While these studies have some value, they have yet to provide a comprehensive, systematic, and dynamic analysis of patents related to the Industrial Internet. Secondly, some studies in related fields have analyzed patent texts or conducted text-based topic extraction. However, conventional methods such as LDA overlook important semantic information, such as word order and syntax, and are unable to capture dynamic modeling. The final research on patent text topic modeling did not incorporate other methods for further analysis.

To address this research gap, the study proposes a research method for analyzing the dynamic evolution of technology using BERTopic and the Hidden Markov Model (HMM). This approach has three advantages. Firstly, the BERTopic model is a deep learning-based topic modeling approach. It can transform text into high-dimensional vectors, capturing semantic information and contextual relationships, and thus extract the technical topics of industrial Internet patents. Secondly, the BERTopic model is a dynamic topic model that can demonstrate the evolution process and trends of technical topics over time. This capability enables us to analyze the developmental stage and direction of technology. Thirdly, a Hidden Markov Model can be constructed based on the time-series data of technology topics obtained from the BERTopic model. By observing the changes in the number of patent applications, it is possible to infer the potential status and likelihood of technological topic transfer. This can help reveal the mechanisms and laws of technological evolution. This paper presents a novel framework for analyzing the dynamic evolution of technology. This framework overcomes the limitations of traditional text mining methods, such as the neglect of deep semantic information and dynamic modeling. It enhances the accuracy and interpretability of technology topic extraction and prediction. This paper utilizes the Derwent Patent Database, which collects 52,121 worldwide industrial Internet patent data from 1965 to 2023. This provides a comprehensive, long-term, and international perspective that broadens the scope and enhances the depth of research on industrial Internet technologies.

The paper is organized as follows: Section 2 reviews the literature on the topic-based patent analytics approach and smart manufacturing. Section 3 introduces the BERTopic and HMM models, as well as theories on Internet governance. Section 4 presents our main findings. Section 5 discusses and analyzes the results. Section 6 concludes the paper.

## 2. Literature review

### 2.1. Industrial Internet patent technology analysis

Most of the previous studies have used patent texts to conduct descriptive and analytical reviews or to extract topics from existing patent documents. However, these studies have some limitations, such as ignoring the dynamic evolution of technology topics and the rich semantic information contained in patent texts. Jian-Qiang Li et al. analyzed the brief history of the Industrial Internet, its architecture, and supporting technologies based on existing literature. They also summarized the application of the Industrial Internet in various fields and discussed the challenges that lie ahead [7] Juite Wang et al. collected patent texts on intelligent manufacturing technology and utilized the Latent Dirichlet Allocation (LDA) method for topic modeling. They constructed three indices, namely the reference rate, claim rate, and scale rate, to evaluate the status of competition analysis within the subject field. In addition, they analyzed the number of patent documents and the growth rate of each topic to determine the level of emerging topics for each subject [8] A comprehensive analysis and overview by Amy J.C. Trappey included basic standards and patents from management standards organizations in the United States, Europe, and China, which host the majority of global manufacturing facilities. They also conducted a comprehensive review of the standards and technologies of the Internet of Things [6]. Lorenzo Ardito et al. collected 61,972 IoT patents filed under the Patent Cooperation Treaty from 2000 to 2012. They examined innovation dynamics and technological evolution by analyzing the time trend of patent applicants, transnational dynamics, and country [5]. Xiang Li et al. utilized bibliometrics to categorize Internet of Things technology into five technical subfields. Based on the De Winter patent database, they used patent metrology to conduct a comprehensive analysis of Internet of Things (IoT) technology and its different technical subfields. They used CiteSpace as an analytical tool to analyze and discuss the innovative characteristics of Internet of Things technology and its various technical subfields [9]. Through a comprehensive review of edge computing in the Industrial Internet of Things, Tie Qiu et al. elaborate on the development and integration process of the Industrial Internet of Things and edge computing. They also proposed a reference architecture for edge computing in the Industrial Internet of Things [10] Li Da Xu et al. reviewed the latest industry-related technologies in the field of Industry 4.0 by analyzing the literature [4]. The existing literature primarily focuses on analyzing patent texts or standards related to the Internet of Things. However, there has been no comprehensive analysis of patent texts specifically related to the Industrial Internet. In addition, the majority of the methods utilized were reviews or topic analyses, without any subsequent analysis of the extracted topics in conjunction with other methods, such as prediction.

### 2.2. Topic model

Topic modeling is a technique that automatically extracts topic information from a large number of documents. The core idea of topic modeling is that each document can be viewed as a mixture of multiple topics, and each topic consists of a set of words. Topic models assist in tasks such as document classification, clustering, and information retrieval. There are three phases in the order of emergence of topic models, as stated by Churchill and Singh [11].

From 1999-2006, the main methods for topic modeling were Latent Semantic Analysis (LSA) using matrix decomposition and Probabilistic Latent Semantic Analysis (PLSA) employing probabilistic graphical modeling. LSA extracts latent semantic information by reducing the size of the document-word matrix using Singular Value Decomposition (SVD) [12]. PLSA represents documents as a mixture distribution of topics and topics as a probability distribution of words, using Maximum Likelihood Estimation (MLE) [13]. LSA and PLSA have the advantage of being able to discover implicit relationships between documents and words. However, they have the disadvantage of ignoring the prior distributions of the words and the document generation process. They also struggle to effectively handle data sparsity and polysemy, and they are prone to triggering overfitting.

From 2006-2011, the primary approaches to topic modeling were latent Dirichlet allocation (LDA) and its extension models derived from probabilistic graphical models. LDA is a generative probabilistic model that introduces Dirichlet prior distributions to regularize the distributions of document-topic and topic-word, following PLSA [14]. The extended model of LDA includes the dynamic topic model, emotional topic model, and other models that incorporate additional information, such as time, space, emotion, and social network. The advantages of Latent Dirichlet Allocation (LDA) and its extensions include preventing overfitting, enhancing the model’s generalization, and incorporating additional semantic and contextual information. The disadvantages include the complexity of the computation and the need to utilize approximate inference methods, such as Gibbs sampling or variational inference [15].

From 2011 to the present, the prevailing approach to topic modeling has been neural network-based topic modeling, as demonstrated by BERTopic. BERTopic is a topic modeling technique that utilizes transformers and c-TF-IDF to create dense clusters. This approach enables the creation of easily interpretable topics while preserving important words in the topic descriptions. BERTopic supports bootstrapping, (semi)supervised, and dynamic topic modeling, as well as LDAvis-like visualization. BERTopic has the advantage of being able to leverage pre-trained language models, which improves the quality and consistency of topics. Additionally, it supports multiple languages and embedded models. However, it has the disadvantage of requiring larger computational resources and storage space [16].

Most topic models are static and cannot be analyzed dynamically. The LDA model is a popular topic model that can extract topics from text. However, the LDA model ignores word order and deeper semantics, such as syntax, and has limited representational capabilities [17]. To effectively address the dynamic nature of technical subjects, this paper utilizes the state-of-the-art BERTopic model and a high-capacity server to handle the extensive computational requirements for modeling.

### 2.3. Hidden Markov Model

A statistical model known as the Hidden Markov Model (HMM), which is an evolution of the Markov model, is capable of describing Markov processes with hidden and unknown parameters. HMM was initially used in speech recognition within the field of natural language processing and genetic analysis in biology. Since then, it has been widely applied in various fields, such as stock prediction and bioinformatics, among others. Notably, it has also been employed in various disciplines related to text, such as literature, bibliography, and patent analysis, among others. Applications of HMM in text analysis generally fall into three categories:

The first category pertains to studies that utilize Hidden Markov Models (HMM) to examine patterns of technological growth and life cycles. These papers typically consider stages of technological growth or life cycle as hidden states, with the number of patents or citation information as observations. Transfer probabilities are calculated based on various assumptions or data sources. For instance, Lee et al. [18] divided the growth of technology into seven stages based on data from patent counts. They employed clustering analysis to examine trends and generate growth probability transition matrices using the Poisson distribution. In this analysis, the growth stages were considered as latent states. Similarly, Hyoung-joo Lee et al. [19] utilized patent citation information to identify latent factors associated with technology growth and knowledge flow. They estimated transfer probabilities by either constructing a patent network or assuming a Poisson distribution. Changyong Lee et al. employed Hidden Markov Models (HMM) to predict the dynamic patterns of technology life cycle stages. They used citation information as individual patent-level observations to generate growth probability transition matrices based on Poisson distributions [20].

The second category encompasses studies that utilize topic models and hidden Markov models (HMMs) to examine the evolution of technological topics. These studies focus on integrating topic models and hidden Markov models (HMMs). In this approach, the extracted topics from the text are used as hidden states. The probability distributions of the topics are treated as observations, and the transition probabilities are determined based on the similarities or co-occurrences of the topics. Wu et al., for instance, extracted topics from thesis data using LDA models and tracked the evolution of topics with HMM. They established hidden states with topics derived from LDA clustering and used the probability distribution of these topics as observations. Word co-occurrence frequencies were used to measure the similarity between topics, which were then utilized as the transfer probability [21]. Wei et al. selected technical topics distilled from LDA as hidden states and observed their probability distributions. They created a co-occurrence normalized matrix for hidden states and used it to calculate transition probabilities. By analyzing the distribution and evolutionary patterns of technological topics using Hidden Markov Models (HMM), the authors identified research and development opportunities in the field of 3D printing [22].

The third category involves studies that apply Hidden Markov Models (HMM) to analyze social media. These papers treat social media comments as hidden states and utilize related indicators as observations to calculate transfer probabilities from different perspectives. For example, Jang employed Latent Dirichlet Allocation (LDA) to model anchored topics from car review data and used Hidden Markov Models (HMM) to forecast hidden topics by considering the number of monthly articles as observations [23]. Suh predicted potential political risks in social media using Hidden Markov Model (HMM) patterns at the observation level. They utilized Natural Language Processing (NLP) to extract latent variables referred to as Political Risk-Related Topics (PRRT) and computed transition probabilities based on energy, sentiment, and social network metrics.

Due to the lack of time-series data specific to the technology itself, existing research on technology development has mainly been conducted by constructing technology life cycles or technology indicator systems. To date, no studies have exclusively analyzed texts related to technology. Given the BERTopic model’s ability to capture temporal data of textual topics, it offers a viable approach for modeling technology topics. Additionally, since the BERTopic model relies on a deep learning-trained vectorized representation of text, it can capture semantic text features more accurately. This, in turn, enhances the precision of the extracted technology topics.

## 3. Methods

### 3.1. BERTopic Model

BERTopic is a topic modeling technique based on deep learning that can extract topics from a large amount of unstructured text and present them in a comprehensible and interpretable manner. Compared to other topic models, such as LDA, this model has the following advantages: it utilizes a pre-trained model to convert the text into a high-dimensional vector. This approach enables the better capture of semantic information and contextual relationships within the text, resulting in more accurate and coherent topics. The c-TF-IDF algorithm is used to cluster text vectors, creating dense topic clusters while preserving important words in topic descriptions. This improves the interpretability and distinguishability of topics. The UMAP algorithm is used to reduce dimensions and visualize the identification of similarities and differences between topics. It also helps to explore and analyze the trend of topic evolution over time, aiding in the exploration and analysis of topics.

BERTopic topic modeling includes the following steps: embedding, dimension reduction, clustering, bag-of-words, and c-TF-IDF. Each step can select an appropriate sample processing method to construct the corresponding topic model.

(1) Document Embedding. Convert the document to a digital format. This paper utilizes the all-MiniLM-L6-v2 word embedding model, which is an English language-based model. It can map sentences or paragraphs to a 384-dimensional vector space and is commonly used in clustering and semantic search tasks.
(2) Dimension reduction. The digital representation has been reduced in size. High-dimensional data poses challenges for clustering models; therefore, dimensionality reduction is necessary. The UMAP method is used for dimensionality reduction in this paper. UMAP is capable of preserving both the local and global structure of the data while reducing its dimension. It has the advantages of strong scalability, fast processing speed, and efficient clustering.
(3) Clustering. Clustering the data after reducing its dimensionality. BERTopic uses the HDBSCAN method for clustering by default. HDBSCAN is a density-based clustering technique that can identify clusters of various shapes and, in certain instances, detect outliers.
(4) Bag-of-words representation. All the documents in a cluster are merged into a single document, and the frequency of each word in the cluster is calculated. This process results in a bag-of-words representation that contains the frequency of each word in each cluster.
(5) Topic representation. The c-TF-IDF method is used to extract topic features. C-TF-IDF is a keyword extraction method based on classified text rather than the entire corpus. It takes into account the impact of feature words on text discrimination and extracts keywords more accurately.

### 3.2. Hidden Markov Models

**Fig 1:**
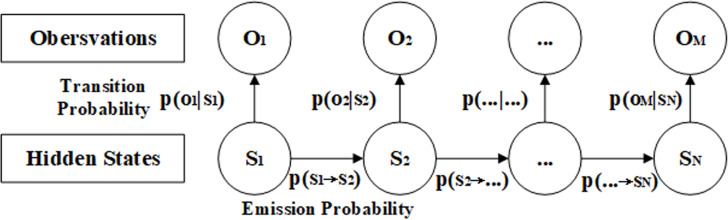
Hidden Markov Models.

Hidden Markov Models (HMMs) are probabilistic models based on time series (see Fig 1). Hidden states and observation states are used to describe the dynamic process and performance of a system. Hidden states are internal states of a system and possess the Markov property, which implies that the current state is solely dependent on the preceding state. Observation states are the outputs of the system and have a probabilistic relationship with the hidden states. Each hidden state can generate an observation state. The parameters of the hidden Markov model include the initially hidden state probability matrix, the hidden state transition probability matrix, and the observation probability matrix. These matrices represent the initial state distribution of the system, the transition law between hidden states, and the generation law for outputs, respectively. The hidden Markov model can be represented by the following five parameters:

(1) Hidden state parameters

HMM has two hidden state parameters: the hidden state and the hidden state sequence. A hidden state is an internal variable that cannot be directly observed. The system exhibits the Markov property, which implies that the current state depends solely on the previous state. The hidden state reflects the internal state and the state transition law of the system. A hidden state sequence is a sequence of state variables with a length of T, representing the dynamic and uncertain internal state of the system at T time points. We use S to denote the set of all possible hidden states, $S=\{s_{1},s_{2},\cdots ,s_{N}\}$, where N is the number of hidden states. We use I to denote a hidden state sequence of length T, $I=\{{\text{i}}_{1},i_{2},\cdots ,i_{T}\}$, where ${\text{i}}_{t}$ is the hidden state at time t, $i_{t}\in S$.

(2) Observational state parameters

The HMM also has two parameters related to the observation state: the observation state and the observation state sequence. The observation state is an output variable that can be directly observed and has a probabilistic relationship with the hidden state. Each hidden state can generate an observation state. The observation state reflects the output and state. The observation state sequence is a sequence of output variables with a length of T. It represents the change process and observability of the system’s output over T time steps. We use O to denote the set of all possible observed states, $O=\{o_{1},o_{2},\cdots ,o_{M}\}$, where M is the number of observed states. We use Q to represent the observation state sequence with length T, $Q=\{{\text{q}}_{1},q_{2},\cdots ,q_{T}\}$, where $q_{t}$ is the observation state at time t, $q_{t}\in O$.

(3) Hidden state transition matrix

The hidden state transition matrix A represents the probability of transitioning from one hidden state to another at any given time point, reflecting the dynamic change pattern of the hidden state. A is the hidden state transition probability matrix: $A={\left[a_{ij}\right]}_{N\times N}$, where $a_{ij}=P(i_{t+1}=s_{j}|i_{t}=s_{i})$, i=1,2,⋯,N, j=1,2,⋯,N, which is the probability of transitioning to the hidden state $s_{j}$ at time t + 1 given that the hidden state $s_{i}$ is at time t.

(4) Observation state probability matrix

The observation state probability matrix B represents the probability of observing a state in a given hidden state at any given time. It reflects the degree of correlation between the hidden state and the output of the observation state. B is the observation state probability matrix: $B={\left[b_{j\left(k\right)}\right]}_{N\times M}$, where $b_{j(k)}=P(q_{t}=o_{k}|i_{t}=s_{j})$, k=1,2,⋯,M, j=1,2,⋯,N. It is the probability of generating the observation state $o_{k}$ under the condition that the time t is in the hidden state $s_{j}$.

(5) Initial hidden state probability matrix

The initial hidden state probability matrix represents the probability distribution of each hidden state at t =  1. The initial state of the hidden Markov chain affects the subsequent state transitions and observations. The setting is the initial hidden state probability vector: $\pi ={\left(\pi_{i}\right)}_{1*N}=(\pi_{1,}\pi_{2,}\cdots \pi_{n})$, where $\pi_{i}=P(i_{1}=s_{i})$, i=1,2,⋯,N. It is the probability of being in the hidden state $s_{i}$ at time t = 1, which is used as the initial value of the hidden state.

The hidden state transition probability matrix A and the initially hidden state probability vector *π* determine the hidden Markov chain and generate an unobservable sequence of hidden states. The observation probability matrix B determines how to generate the observed state from the hidden state, and combines with the hidden state sequence to determine how to generate the observation sequence.

### 3.3. Internet governance theory

The five major components of Internet governance include stakeholders, resources, regulations, principles, and outcomes. These components can also be summarized as the subject, object, and basic means [24]. This paper primarily focuses on analyzing the future development trends of industrial Internet technology using the theory of Internet governance. Internet governance objects refer to the physical layer, logical layer, data layer, and interaction layer [6]. The physical layer is the foundational infrastructure layer of the Internet, encompassing servers, storage, fiber optic cables, and other hardware components. The logical layer refers to the technical layer of the Internet, which is constructed with TCP and IP as the core protocols, along with hardware and interface standards. The data layer is the content layer of the Internet. It refers to the internet content stored in the physical layer and transmitted through the logical layer. This includes text, pictures, audio, and video that are displayed. The interaction layer refers to the behavior of people on the Internet, which is based on the application of the content carried by the fundamental resources of the Internet [25]. Among these four layers, the physical layer and the logical layer play the roles of carrying data transmission and facilitating interaction on the internet. However, behind these “physical” factors, the dominant role is played by human behavior. Therefore, the research focus of Internet governance is on the data layer and the interaction layer. This includes studying the behavior of individuals who use the internet to create, transmit, and access content. This study focuses on the theory of Internet governance to analyze the future development trends of industrial Internet technology.

The main components of the Internet governance model primarily consist of three sectors: government, private sector (including companies, private studios, etc.), and civil society. This division is based on the work report of the United Nations Internet Governance Working Group. The means of Internet governance models mainly include four aspects: legal norms, administrative measures, self-discipline management, and technical control. Legal norms are an important component of Internet governance models, as they regulate Internet behavior. Administrative means are the conventional methods often used by the government. Self-discipline management is divided into two parts: industry self-discipline and netizen self-discipline. Technical control mainly involves controlling and ensuring the integrity of the data layer and the presentation layer.

## 4. Illustration

### 4.1. Research framework

**Fig 2:**
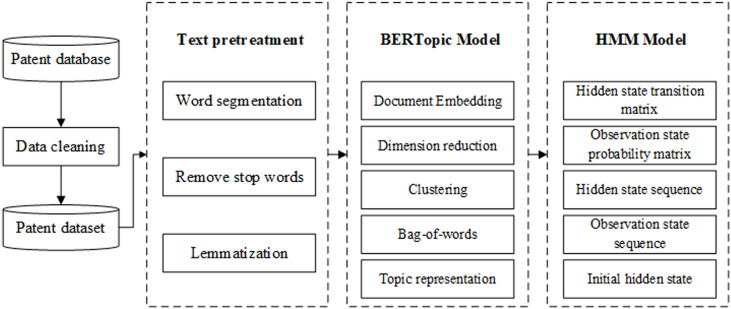
Overall framework.

Firstly, the Derwent Patent Database is used to gather industrial Internet patent data from 1965 to 2023. Subsequently, the data is cleaned to obtain the dataset for this study. Secondly, data preprocessing is performed on the dataset, and the BERTopic model is applied to extract topics related to patent technology. This allows for the analysis of key technologies in the field of the industrial Internet. According to the object theory of Internet governance, patent technology is classified. These classified topics are used as the hidden state in the Hidden Markov Model. The number of patent applications serves as an observational indicator for predicting the evolutionary trends of patent technology topics and exploring the mechanisms behind the evolution of industrial Internet technology (see Fig 2).

### 4.2. Dataset

#### 4.2.1. Text acquisition and data description.

This study utilizes the Derwent International Patent Database as the data source and analyzes technological advancements in the field of the industrial Internet using patent data. The term “Industrial Internet” refers to the seamless integration of advanced information technology with the manufacturing industry. Different countries have varying definitions of “it” but the fundamental content is generally similar. According to the key terms, core architecture, and key technologies of the industrial Internet [3], this study uses the following search terms to retrieve patent data related to the industrial Internet [26]: ‘industry internet’, ‘industry internet of things’, ‘IIoT’, ‘industrial IoT’, ‘industrial platform’, ‘industrial cybersecurity’, ‘industrial big data’, ‘industrial artificial intelligence’, ‘industrial cloud’ [27]. Finally, this study obtained 52121 patent data from October 1965 to October 2023 (see S1 Table).

#### 4.2.2. Text data preprocessing.

This study preprocessed the English text for topic modeling. The preprocessing steps included removing stop words using the English stop word library in the NLTK package, tagging the parts of speech such as nouns, verbs, adjectives, etc. in the text using the WordNet Lemmatizer package for lemmatization, and then converting the text to lowercase. These steps improved the clarity and consistency of the text, which facilitated the extraction of the main topic.

#### 4.2.3. The optimal number of topics.

**Fig 3:**
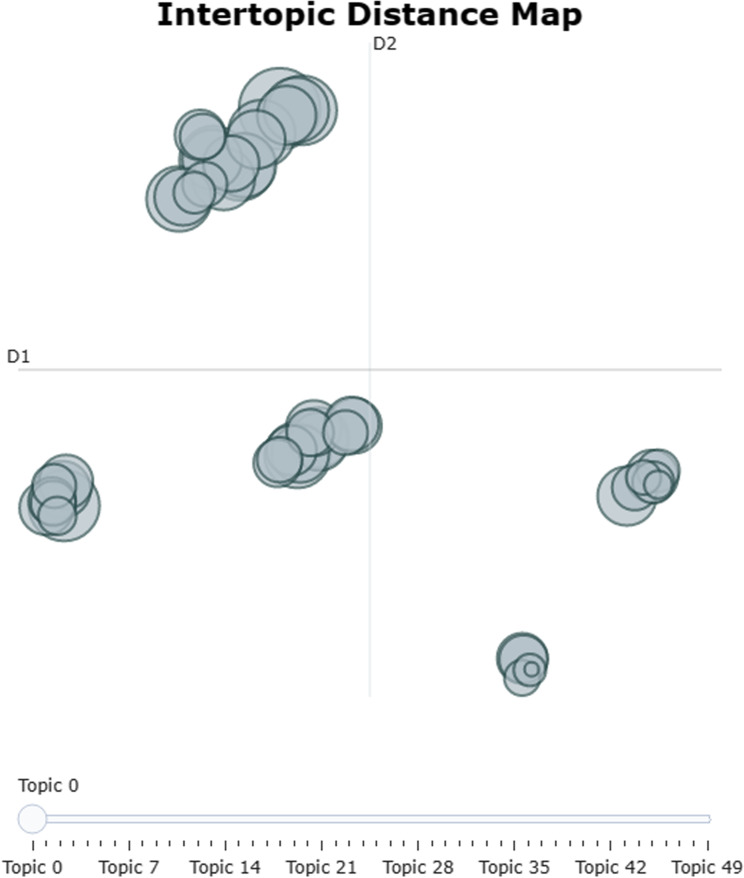
Clustering of 50 topics.

Unlike LDA, which selects the optimal number of topics based on the perplexity and coherence index, the BERTopic model does not generate a perplexity or coherence curve. There are three approaches to determining the number of topics: automatic generation, manual specification, and deletion based on topic clustering. To ensure that no important topics were overlooked, the initial number of topics was set to 50 (see Fig 3), and then these topics were merged [28]. Finally, there were five patent topics.

### 4.3. Overview of industrial Internet patents

**Fig 4:**
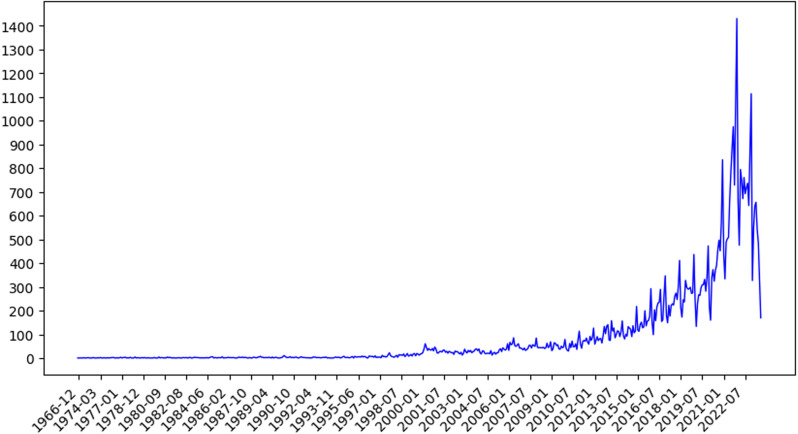
The number of patent applications in the field of industrial Internet from 1965 to 2023.

Industrial Internet patent data from October 1965 to October 2023 was collected in this study, as shown in Fig 4. It can be seen from the figure that the development of the Industrial Internet was in a stable stage before 1999. The number of patent applications in the 34 years accounted for only 2.18% of the total, which is a very low proportion. After 1999, the development of the industrial Internet entered a stage of slow growth, which then transitioned to rapid growth. During this period, the number of patent applications exhibited a gradual increase, eventually leading to an exponential rise. This indicates that the technological innovation of the Industrial Internet experienced a gradual increase until 1999, after which it experienced a significant rise.

### 4.4. Industrial Internet technology topic extraction

To extract the key technical topics from the extensive patent texts, the study followed the steps outlined below. Firstly, the BERTopic model utilized the all-MiniLM-L6-v2 model, which is specifically trained for English text, to convert the text into a digital format. The sentences and paragraphs were then mapped to a dense vector space with 384 dimensions. The UMAP method was then used to map the data from the previously generated high-dimensional space to a low-dimensional space. This was done to reduce dimensionality while preserving the relationships and structure among data points. Secondly, the CountVectorizer function was used to convert the text into a matrix of word frequencies. Each row of the matrix represents a sentence, and each column represents a word. The improved c-TF-IDF method, which is based on TF-IDF, was used to extract the word frequency based on class. Finally, the processed data was visualized.

**Fig 5:**
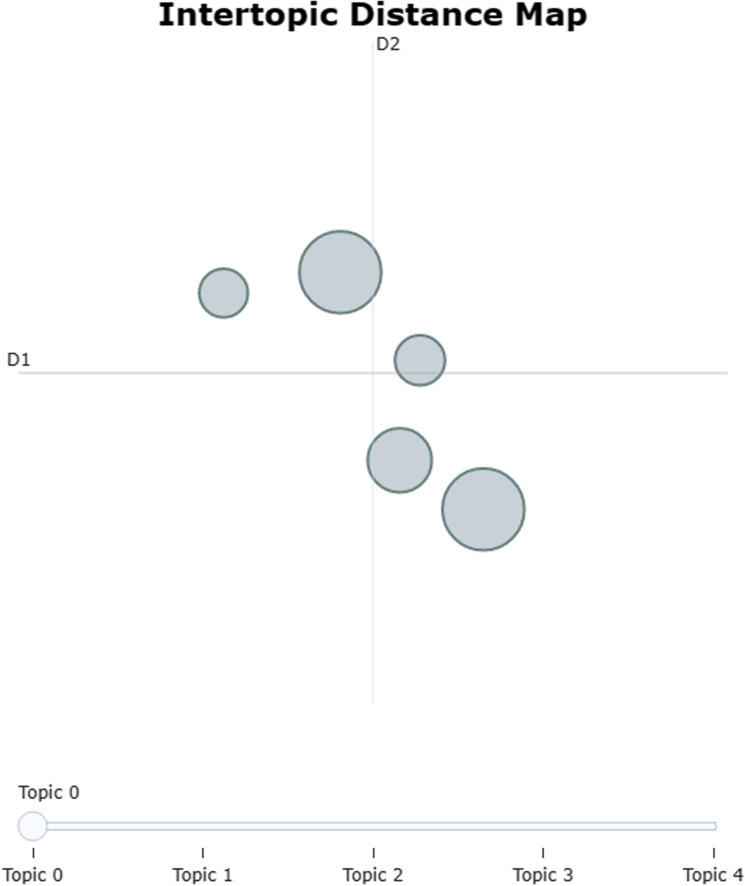
Industrial Internet patent topic.

**Fig 6:**
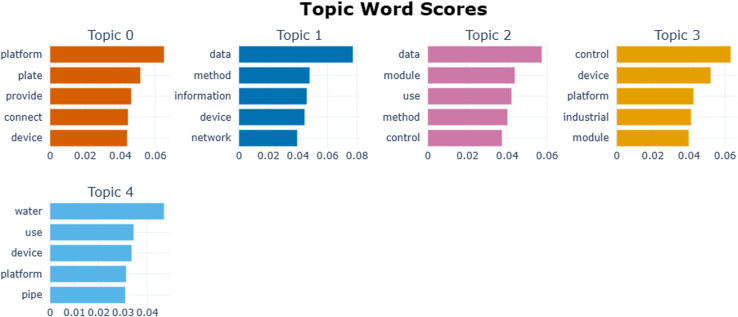
Patent subject word frequency distribution.

The BERTopic topic model was used to analyze the topics in the abstract text of industrial Internet patents, and several topics obtained are shown in Fig 5. Finally, there were five patent topics (see Fig 5), and the keywords and their frequencies for each topic are shown in Fig 6.

This study took the following steps to ensure the accuracy and consistency of the topics: First, two doctoral students in the field of management collaborated to analyze the topic terms. For topics that have ambiguities, a new doctoral student was introduced to discuss and analyze the results together. Second, expert interviews were conducted to gather opinions until a final consensus was reached. Lastly, according to the categorization of objects in the theory of Internet governance, the topic terms were divided into four aspects: the physical layer, the logical layer, the data layer, and the interaction layer [29].

After completing the aforementioned steps, this study identified four main areas of focus for industrial Internet patents (see Table 1). These areas include: (1) Equipment interconnection platform (Topic 0), and liquid gas storage monitoring device (Topic 4) in the physical layer. (2) The logical layer includes the remote-control system for industrial equipment (Topic1_3).

**Table 1:** Patent topic keyword.

Governance object	Topic	No.	Keywords
Physical layer	Equipment interconnection platform	Topic 0	platform, plate, provide, connect, device, end, mechanism, support, frame, rod
Data layer	Data processing and information services	Topic 1	data, method, information, device, network, use, include, service, industrial, user
Interactive layer	Modular image processing and control method	Topic 2	data, module, use, method, control, device, industrial, image, include, point
Logical layer	Industrial equipment remote control system	Topic 3	control, device, platform, industrial, module, connect, camera, power, laser, use
Physical layer	Liquid gas storage monitoring device	Topic 4	water, use, device, platform, pipe, provide, material, tank, air, gas

The equipment interconnection platform (Topic 0) serves as a cornerstone of the Industrial Internet [30]. It enables intelligent management of devices and secure data flow through cloud-based connectivity, thereby enhancing industrial production efficiency, quality, and innovation [31]. This platform is instrumental in four critical applications. First, it facilitates remote monitoring and maintenance of equipment, which reduces labor costs and increases equipment reliability and availability [32]. Second, it supports cooperative operations, flexible production, and intelligent scheduling to enhance production flexibility and efficiency [33]. Third, it collects and analyzes equipment data to improve product quality and performance. This is exemplified by the deployment of a 5G + 8K surface inspection system in the steel industry, which has improved defect detection [34]. Fourth, it enables the intelligent upgrading of equipment, which leads to the development of new products and services. This includes value-added services for equipment management, preventive maintenance, and personalized customization based on networked products [35].

Liquid gas storage monitoring devices (Topic 4) utilize wireless sensor networks and cloud computing technology to enable real-time collection, transmission, analysis, and control of various parameters of liquid gas storage tanks. This allows for remote monitoring and management of liquid gas [36]. This technology has extensive applications in fields such as medicine, chemistry, and food. It optimizes the supply and utilization of liquefied gas in the chemical industry, thereby improving production efficiency and reducing energy consumption and emissions [37]. In the medical field, it ensures the quality and safety of liquefied gases, preventing shortages and waste, thereby improving medical service levels and effectiveness [38]. The food industry, regulates the consumption and loss of liquids and gases, thereby improving food quality and safety and extending shelf life [39].

The industrial equipment remote control system (Topic 3) is a technology that utilizes communication technology and artificial intelligence to gather, analyze, and manage industrial equipment and systems in various locations in real-time. This technology has three main applications: industrial automation, industrial robotics, and industrial IoT [40]. In industrial automation, this technology enables remote monitoring and control to enhance efficiency and quality, reduce consumption, enable remote upgrading and modification, and improve functionality and performance [41]. In industrial robotics, this technology extends the application scope and scenarios, promotes remote collaboration and learning, and enhances intelligence and efficiency [42]. In industrial IoT, this technology supports remote monitoring and control, provides visualization and intelligent support, enhances monitoring and management capabilities, enables remote optimization and tuning, and fosters innovation and value [43].

Data processing and information services (Topic 1) refer to the utilization of cloud computing, big data, artificial intelligence, and other technologies to store, analyze, and extract value from vast amounts of data generated by industrial equipment and systems. This concept is commonly applied in various areas, including industrial intelligence, industrial safety, and industrial services. In the field of industrial intelligence, these technologies enable intelligent analysis and mining of industrial data, enabling informed decision-making and control of industrial systems. Furthermore, they enable intelligent prediction and recommendation of industrial data, as well as intelligent optimization and adjustment of industrial systems [43]. In the realm of industrial security, these technologies contribute to the secure storage and protection of industrial data, thereby enhancing the security and reliability of industrial systems. They also enable security monitoring and early warning for industrial data, thereby improving security prevention and emergency response capabilities [44]. In the field of industrial services, the use of these technologies allows for the servitization and commercialization of industrial data, thereby improving the functionality and value of industrial systems. It also promotes service innovation and new service models for industrial data, thereby improving service quality and effectiveness [45].

The modular image processing and control method (Topic 2) utilizes image processing technology and control theory to enable real-time acquisition, analysis, and control of image information for industrial equipment and systems. This technology is widely used in areas such as industrial inspection, industrial robotics, and industrial vision. In industrial inspection, these methods enable the automatic detection and identification of surface defects, as well as the measurement of size, shape, color, and other characteristics of industrial products. This helps improve the quality and consistency of industrial products while reducing the errors and costs associated with manual inspection [3]. In the field of industrial robotics, these methods are utilized to automatically control and optimize functions such as vision navigation, localization, tracking, grasping, and other tasks performed by industrial robots. This increases the flexibility and accuracy of industrial robots, expanding their range of applications and scenarios [46]. In industrial vision, these techniques are used to collect, transmit, store, analyze, and apply image information from industrial scenes in real-time. This helps visualize and enhance the efficiency of industrial systems. It also improves their monitoring and management capabilities [47].

### 4.5. Analysis of the evolution mechanism of hot technology in industrial Internet

**Fig 7:**
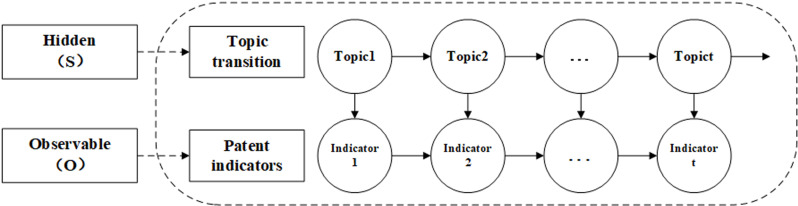
Hidden Markov Model based on patent topic evolution.

The BERTopic model was applied to the patents to extract five topics. These topics were then classified into four categories based on the theory of Internet governance objects. The topics in the same category were merged, resulting in four reclassified topics: device interconnection platform and liquid gas storage monitoring device for the physical layer (Topic 0 and Topic 4), industrial equipment remote control system for the logic layer (Topic 3), data processing and information service for the data layer (Topic 1), and modular image processing and control method for the interaction layer (Topic 2). These reclassified topics were used as the hidden state, while the monthly number of patent applications was used as the observation state in a hidden Markov model with continuous observations. The hidden topic sequence was then predicted based on the annual number of patent applications using the hidden Markov model (see Fig 7).

#### 4.5.1. HMM model parameters.

**Fig 8:**
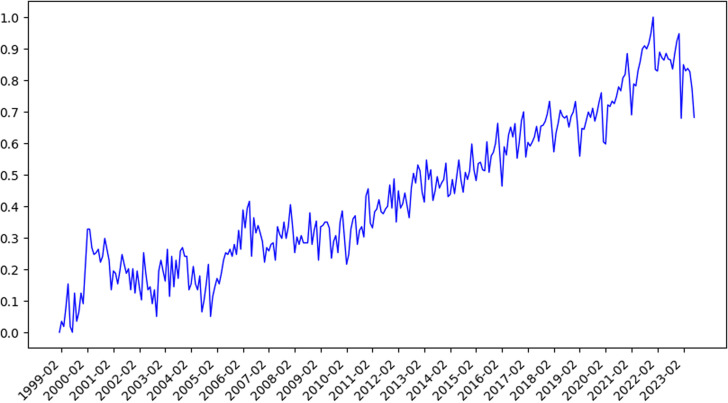
Observation sequence diagram.

The five parameters of HMM were determined according to the definition of HMM in Section 3.2. The maximum likelihood method was used to estimate the parameter values. The hidden state S consisted of the four reclassified topics, which were grouped according to the theory of industrial Internet governance. The initial probability of the hidden state was a uniform distribution [23]. The topics with the highest proportion of patent applications in each period and the highest frequency of topic transitions in the adjacent periods were counted. The transition frequency matrix was obtained and normalized by row to obtain the hidden state transition probability matrix A (Table 2). The patent data was divided into monthly periods, and the number of patent applications per month was used as the observation. The patent data did not fluctuate for a long time in the early years, which would affect the HMM calculation. Therefore, the data with fluctuations since 1999 was selected as the starting point for prediction [23], following the existing literature. A total of 297 months of patent application data were obtained and logarithmically normalized using min-max normalization. This was done to satisfy the HMM assumptions and to minimize the impact of data fluctuations on the prediction. The range of observations at each time step was between 0 and 1. The observation sequence Q was obtained (see Fig 8).

**Table 2:** Hidden Markov hidden transition probability moment.

	Physical layer	Logic layer	Data layer	Interaction layer
Physical layer	0.667	0.008	0.317	0.008
Logic layer	0.500	0.000	0.500	0.000
Data layer	0.216	0.005	0.763	0.016
Interaction layer	0.333	0.000	0.667	0.000

#### 4.5.2. HMM model calculation.

**Fig 9:**
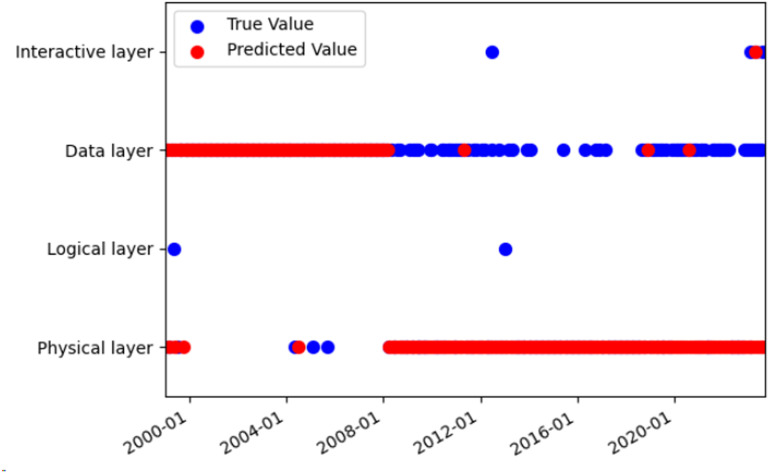
Comparison of hidden state prediction based on the Viterbi algorithm.

**Fig 10:**
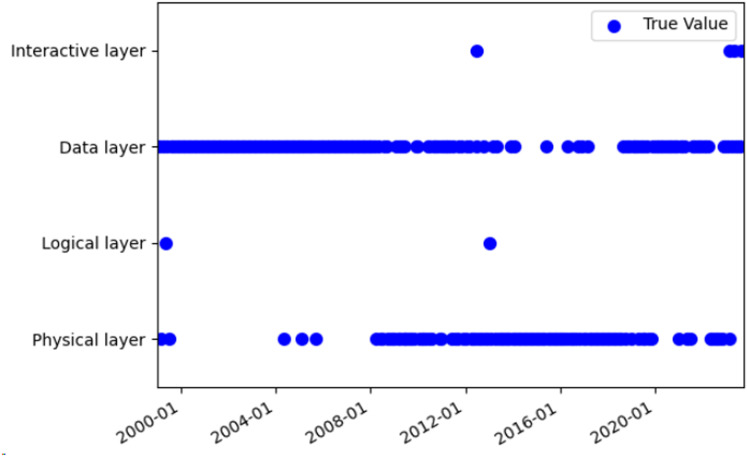
The true value of the hidden state based on Viterbi algorithm.

**Fig 11:**
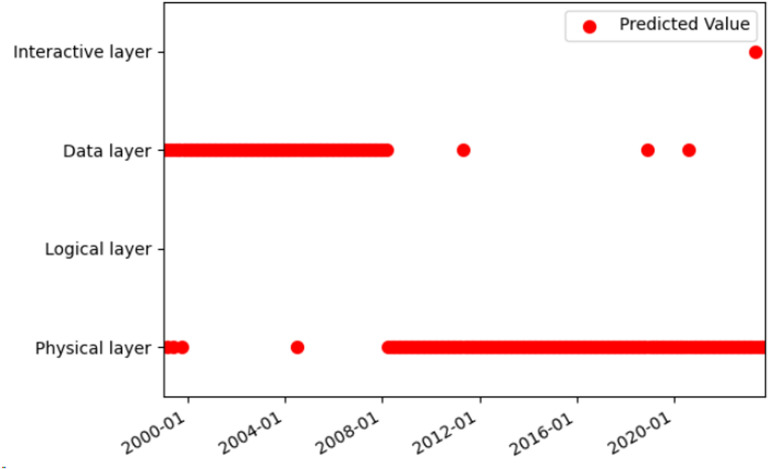
The predicted value of the hidden state based on Viterbi algorithm.

The HMM prediction problem was solved by the Viterbi algorithm, which utilizes dynamic programming to identify the optimal path with the highest probability among the potential sequences of hidden states. The Viterbi algorithm was applied to predict the hidden states, and the predicted hidden states were compared with the actual hidden states (see Figs 9–11). The prediction accuracy was 74.75%, which was higher than the 60% reported in a previous study [23] for the same type of data and model. The reason for the difficulty in achieving a higher accuracy than 0.9 [23] was that the topic model merged the text of some small datasets each month, resulting in data loss.

## 5. Discussion

### 5.1. Technology topic development trend

**Fig 12:**
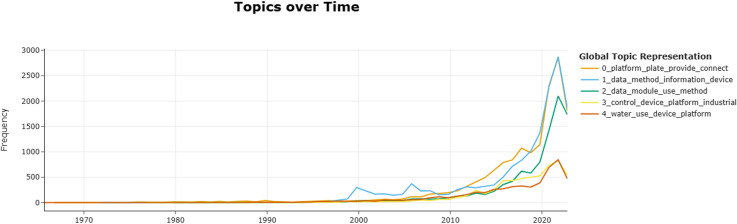
The development trend of industrial Internet patent technology according to the year division.

Five topics were extracted from the patents using the BERTopic model. The topics were analyzed by year to reveal the technology trends more clearly (see Fig 12). The technology trends were divided into three phases based on their characteristics. The following were as follows:

Phase 1 (1965–1998): The Industrial Automation Phase. This phase spanned 33 years, accounting for 56.9% of the total time. The number of patent applications in this period accounted for approximately 2.18% of the total. The development of Internet technology was relatively smooth and unchanged. This stage was characterized by the third industrial revolution, which involved the adoption of sensors, actuators, robots, and other intelligent components and devices, as well as automated control systems. They enable automated control and data acquisition of industrial equipment, improving the flexibility and reliability of industrial production, and enhancing the management and optimization capabilities of industrial processes [48].

Phase 2 (1999–2021): The Industrial Digitalization Phase. The development trend of this phase showed a gradual upward trajectory. The number of patent applications has increased significantly. The development of industrial internet technology has entered a rapid phase. This phase marked the fourth industrial revolution, which was driven by the Internet of Things (IoT) and the Industrial Internet. The development of data processing and information services (Topic 1) started earlier, reaching a small peak in 2000, and remained at a high stage for the next 10 years. Device interconnection platform (Topic 0) technology emerged later but became the leading technology in development. Modular image processing and control methods (Topic 2) had smooth development in the early stages but entered a rapid growth stage after 2020. Remote control systems for industrial equipment (Topic 3) and liquid and gas storage and monitoring devices (Topic 4) had steady development trends. The IoT paradigm was first introduced by Kevin Ashton in 1998 as a concept for connecting things or objects to the Internet. The concept of the industrial Internet was originally proposed by General Electric (GE) in 2012. It involves the integrated application of big data analytics and remote control technologies based on the Internet of Things (IoT). The goal is to optimize the operation and maintenance of industrial facilities and machines as well as improve the operational performance of assets. In July 2016, the International Society of Automation (ISA), the Process Control and Safety Forum (PCS) in Houston, Texas, and the ISA’s communications department convened a panel discussion to focus on and discuss the Industrial Internet of Things (IIoT) [49]. This was followed by a relatively rapid development of the industrial Internet of Things (IoT).

Phase 3 (2022-2023): The trend in this phase declined. The number of patent applications has dropped. China accounted for approximately half of the global patent applications in the field of industrial Internet. China recently raised the threshold for patent applications to enhance the quality of patents. The overall number of patent applications declined [50].

### 5.2. The evolution of the technical topic

The HMM model was used to investigate the evolutionary mechanism of industrial internet technology. The transition matrix of the HMM model reveals that the highest transition probabilities occur between the data layer to data layer, interaction layer to data layer, and physical layer to physical layer, with values of 0.763, 0.667, and 0.667, respectively. The trends of these three types of transitions were analyzed and compared with related reports to validate the accuracy of the results.

The transition from one data layer to another indicates that data layer technology is a research hotspot in the field of the industrial internet. As shown in Fig 11, data layer technology was a prominent topic throughout all stages of development. The evolution of data layer technology has involved a transition from a low level to a high level, from simplicity to complexity, and from singularity to multiplicity. The evolution from low-level to high-level started with basic data collection and storage advanced to complex data processing and analysis, and finally reached intelligent data visualization and application. The data layer technology has been continuously upgraded and optimized. The evolution from simplicity to complexity began with a single data type and format, then expanded to include multiple data types and formats, and ultimately advanced to encompass the fusion and sharing of diverse data. The data layer technology has continuously evolved and become more complex. The evolution from single to multiple data applications started with single-point data applications, progressed to multi-point data applications, and eventually expanded to network data applications. The data layer technology has continuously expanded and diversified. This might be driven by the following factors: First, the demand-driven industrial internet requires more data to support intelligent decision-making and control of industrial production. This improves production efficiency and quality while reducing resource consumption and environmental impact [51]. Secondly, the technology-driven industrial internet has enhanced technical expertise and capabilities for collecting, transmitting, storing, managing, analyzing, and applying data. This has enabled the comprehensive, in-depth, and extensive use of data [52]. The policy-driven industrial internet has improved the policy environment and standard system of the industrial internet, thereby promoting the openness, sharing, and collaboration of data [53].

The transition from the interaction layer to the data layer can be influenced by various factors. Firstly, the application-driven industrial internet requires a larger volume of data to achieve interconnection, interoperability, and interaction within the industrial system. This, in turn, enhances the synergy and flexibility of the industrial system [54]. Secondly, the user-driven industrial internet requires more data to fulfill the personalized and customized needs of its users, thereby enhancing user satisfaction and loyalty [55]. The innovation-driven industrial internet requires more data to support the development and promotion of new products and services, thereby enhancing the competitiveness and impact of the industrial internet [7].

The transition from one physical layer to another, which ranks third in terms of significance, indicates ongoing innovation and progress in physical layer technology. As shown in Fig 11, physical layer technology emerged as the most popular topic in the second and third stages, with a higher frequency. The physical layer technology includes 5G, 6G, edge computing, software-defined networking, network slicing, blockchain, and more. These technologies have addressed the issues of network congestion, delay, interference, and attacks, while also meeting the demands for real-time data, reliability, and security in industrial settings. They improved the performance and efficiency of the industrial internet [56]. The development of physical layer technology has undergone the following evolution: It started with the development of fiber optic communication technology, followed by wireless communication technology, 5G technology, and satellite communication technology. The communication mode was continuously innovated and optimized. The evolution from single-point to multi-point communication technology began with single-point communication technology and progressed to multi-point communication technology, and eventually to network communication technology. The communication range was continuously expanded and diversified. The evolution from single to diversified sensing technology began with a limited measurement range. It then developed into diversified sensing technology capable of measuring a variety of things and finally advanced to intelligent sensing technology. The sensing function has been continuously improved and enhanced. The evolution from passive control technology to active control technology, and finally to autonomous control technology, has occurred. The control mode was continuously innovated and optimized. The technical applications of the Industrial Internet have been continuously expanded and deepened, encompassing various fields including manufacturing, energy, agriculture, transportation, and medical care [57].

## 6. Conclusion

The Derwent Innovations Index was used to collect patent application data from around the world between 1965 and 2023. The BERTopic method was applied to extract the topic of industrial internet patent technology. The extracted topics were then reclassified according to the theory of Internet governance. The reclassified topic was used as the hidden state in the hidden Markov model, while the number of patent applications served as the observation. The hidden Markov model predicted potential technical topics and explored their potential evolutionary mechanisms. The main conclusions of this study were as follows: The development of the world’s industrial internet technology can be categorized into five main types, which align with four categories of Internet governance theory. The physical layer category includes device interconnection platforms and devices for monitoring liquid and gas storage. The logic layer category includes the remote-control system for industrial equipment. The data layer category includes data processing and information services. The interaction layer category includes modular image processing and control methods. The data layer technology underwent the greatest change, followed by the physical layer technology.

### 6.1. Implication

This study proposes a novel approach that combines the BERTopic model with the hidden Markov model and incorporates Internet governance theory to develop a framework for analyzing the dynamic evolution in the field. This hybrid approach overcomes the limitations of traditional text mining techniques, thereby enhancing the accuracy and interpretability of technology topic extraction and prediction. Moreover, the study collects a comprehensive dataset of global industrial Internet patent data from 1965 to 2023, utilizing the Derwent Patent Database. This extensive dataset provides a long-term, comprehensive, and international perspective on research in industrial Internet technology, thereby enhancing the scope and depth of the study.

The paper also provides managerial implications by uncovering the evolutionary patterns and underlying mechanisms of industrial Internet technology. This information is of considerable value to both corporate and government entities, as it provides insights that can help them effectively understand trends and opportunities in technological development. This, in turn, enhances the efficiency and impact of their innovation efforts. Additionally, the study presents an analysis methodology based on patent data that proves to be effective in identifying the key technologies within the field of the Industrial Internet. Future enterprises can utilize this approach to predict emerging technology trends based on the number of patent applications. This, in turn, provides a valuable reference for devising strategies and policies for technological innovation.

### 6.2. Limitation

This study explores the extraction and prediction of topics related to industrial Internet technology using patent data as the primary source. However, it is essential to acknowledge the limitations of the study, which could benefit from further improvement and refinement. Firstly, the paper solely relies on patent data, neglecting other forms of data such as literature and software works. This may result in a lack of diversity in technology types and an incomplete representation of the overall development of the industrial Internet industry. Future research can address this by expanding the types of data and incorporating diverse data sources to improve data coverage and representativeness. The study, secondly, utilizes a first-order hidden Markov model, neglecting to account for the intricate connections among hidden states. This could lead to a reduction in prediction accuracy and an inability to effectively uncover the mechanism of the technology’s evolution. Subsequent research can explore higher-order hidden Markov models or combine the model with other techniques to enhance its expressive and fitting capabilities.

## Supporting information

S1 Table Supporting information. This table provides the raw data collected during the study.
(XLSX)
